# Effect of NaCl Concentration on Microbiological Properties in NaCl Assistant Anaerobic Fermentation: Hydrolase Activity and Microbial Community Distribution

**DOI:** 10.3389/fmicb.2020.589222

**Published:** 2020-10-09

**Authors:** Heliang Pang, Xiaodong Xin, Junguo He, Baihui Cui, Dabin Guo, Shiming Liu, Zhongsen Yan, Chong Liu, Xinyu Wang, Jun Nan

**Affiliations:** ^1^School of Environmental Science and Engineering, Huazhong University of Science and Technology, Wuhan, China; ^2^School of Environment, Harbin Institute of Technology, Harbin, China; ^3^Department of Environmental Science and Engineering, Huaqiao University, Xiamen, China; ^4^School of Civil Engineering, Guangzhou University, Guangzhou, China; ^5^Advanced Environmental Biotechnology Centre, Nanyang Environment and Water Research Institute, Nanyang Technological University, Singapore, Singapore; ^6^College of Civil Engineering, Fuzhou University, Fuzhou, China; ^7^Frog Biotechnology Co., LTD, Harbin, China

**Keywords:** sodium chloride, anaerobic fermentation, hydrolase activity, waste activated sludge, microbial community

## Abstract

Previous studies have demonstrated that sludge hydrolysis and short-chain fatty acids (SCFAs) production were improved through NaCl assistant anaerobic fermentation. However, the effect of NaCl concentrations on hydrolase activity and microbial community structure was rarely reported. In this study, it was found that α-glucosidase activity and some carbohydrate-degrading bacteria were inhibited in NaCl tests, owing to their vulnerability to high NaCl concentration. Correspondingly, the microbial community richness and diversity were reduced compared with the control test, while the evenness was not affected by NaCl concentration. By contrast, the protease activity was increased in the presence of NaCl and reached the highest activity at the NaCl concentration of 20 g/L. The protein-degrading and SCFAs-producing bacteria (e.g., *Clostridium algidicarnis* and *Proteiniclasticum*) were enriched in the presence of NaCl, which were salt-tolerant.

## Introduction

It is estimated that China’s total waste activated sludge (WAS) production will reach 60 million tons by 2020 (Li et al., 2018). The disposal of WAS has become a global problem. The yield of WAS had reached or even exceeded the environmental load, which increased environmental risks and disposal costs (Sun et al., 2015; Chen et al., 2019; He et al., 2019; Zhang et al., 2019). In recent years, carbon recovery in anaerobic fermentation has attracted wide attention (Cheng et al., 2020; Pang et al., 2020d). Through anaerobic fermentation process, biodegradable organic matter in WAS can be converted into short-chain fatty acids (SCFA), which was a promising carbon source with a wide range of applications (Zhang et al., 2018, 2020; Xin et al., 2020). For example, it could be used as an external carbon source to supplement the carbon gap in wastewater treatment plants (WWTPs) and to promote the biological production of electricity/polyhydroxyalkanoate (PHA; Cai et al., 2009; Li et al., 2011; Chen et al., 2013). Generally, hydrolysis and acidification were involved in anaerobic fermentation for SCFAs production (Luo et al., 2019). The hydrolysis step was regarded as the rate-limiting stage, as the biodegradable organic matters were wrapped in WAS flocs, which reduced their usability for microorganism (Lv et al., 2010). As such, WAS solubilization with the aim of organic matter release was necessary for improving anaerobic fermentation efficiency.

NaCl (sodium chloride) is an inexpensive chemical with a wide range of sources. It has been reported that high NaCl concentration could induce sludge solubilization and deteriorate floc structure (Reid et al., 2006; Cui et al., 2015; Su et al., 2016; Lu et al., 2019). In our previous research, a novel and efficient NaCl assistant anaerobic fermentation strategy was developed for bio-production of SCFAs (Pang et al., 2020e). In the process of NaCl assistant anaerobic fermentation, the addition of NaCl resulted in significant osmotic pressure difference between the sludge phase and the liquid phase, which caused the decomposition of WAS flocs and the breakdown of extracellular polymeric substances (EPS). As such, the sludge flocs were dissolved with release of biodegradable organic matter. Thereby, the efficiencies of sludge hydrolysis and the subsequent SCFAs production were improved. It was reported that numerous soluble chemical oxygen demand (SCOD) of 4,092 mg/L was released into the supernatant at the optimal NaCl concentration of 20 g/L, meanwhile considerable SCFAs of 288.2 mg COD/g VSS was produced through a 4-day anaerobic fermentation (Pang et al., 2020e). Such performances on sludge solubilization and SCFAs yield in NaCl assistant anaerobic fermentation were comparable to the anaerobic fermentation with chemical pretreatments (e.g., surfactants, enzymes, cation-exchange resin, etc.; Huang et al., 2015; He et al., 2018; Pang et al., 2020a,b, 2021).

Furthermore, numerous NaCl was remained in the fermented sludge, which was feasible for reuse once the produced SCFAs could be utilized and consumed. As declared in previous study, the NaCl assistant anaerobic fermentation was indeed a green and efficient approach with some advantages, e.g., none irresistibly consumed chemicals, considerably reduced treatment costs and avoided environmental risks of remained NaCl. After NaCl assistant anaerobic fermentation, the fermentative liquid with numerous SCFAs could be used as external carbon source, e.g., the substrate for biological electricity production through microbial fuel cell (MFC) or the external carbon source for supplementing the carbon gap in municipal wastewater. The feasibility of these utilization approaches were discussed in our previous study (Pang et al., 2020e). After utilization of the soluble organic matters, the fermentative liquid could be reused for NaCl assistant anaerobic fermentation since there is numerous NaCl existed in the fermentative liquid. In this way, the treatment agent (i.e., NaCl) could be recycled, implying that considerable chemical agent and treatment costs could be saved.

Despite the sludge solubilization, EPS disruption, SCFA yield, and relevant mechanism were explored in our previous study (Pang et al., 2020e); the effects of NaCl concentration on enzyme activity and microbial community structure are still unfathomed. Actually, the tolerance of bacteria for high salinity condition is different. The harsh condition of high NaCl concentration might not be conducive to the survival of some microorganism in WAS, which was related to microbial community structure and might lead to shift of dominant bacterial community (Duan et al., 2016). In ecosystem, the functional bacteria distribution is critical to the system functions (Xin et al., 2015; Xu et al., 2018, 2019; Ji et al., 2020). In the anaerobic fermentation process, both the biodegradation of dissolved organic matters (DOMs) and the consumption of SCFAs mainly rely on appropriate microbial communities. As such, exploring the hydrolase activity and microbial community structure under different NaCl concentrations were beneficial to completely understand the NaCl assistant anaerobic fermentation process and optimize the process parameters. Furthermore, the evolutions of microbial community structure and metabolic activity at different NaCl concentrations during anaerobic fermentation process have rarely attracted our attention. Under this circumstance, the functional characteristics of the sludge ecosystem and the biodegradation pathway of DOMs were seriously affected, which might be varying at different NaCl concentrations and be dissimilar to that in conventional anaerobic fermentation (without NaCl addition). The microbial and enzymic characteristics of sludge related to different NaCl concentration in NaCl assistant anaerobic fermentation might be an interesting issue.

In this study, the microbiological characteristics at different NaCl concentrations were explored in the anaerobic fermentation system. The main objectives of this research include (1) investigating the effect of NaCl concentrations on hydrolase activity in anaerobic fermentation process; (2) exploring the microbial community structure and identifying main species at different NaCl concentrations; and (3) analyzing biodegradation pathway of DOMs for SCFAs accumulation in NaCl assistant anaerobic fermentation process. Through this research work, we can further understand the metabolic activity and microbial community structure related to different NaCl concentrations during anaerobic fermentation, which improved the knowledge of community ecology.

## Materials and Methods

### Characteristics of Thickened WAS

The WAS samples were obtained from a municipal WWTP locating in Harbin city, Heilongjiang province, China. The collected WAS samples were thickened at 4°C for 12 h, which was then used for the anaerobic fermentation experiments. The total suspended solids (TSS), volatile suspended solids (VSS), and total chemical oxygen demand (TCOD) of the thickened WAS were 19.2 ± 0.2, 13.2 ± 0.1, and 17.1 ± 0.3 g/L, respectively. The sludge pH was 6.95 ± 0.15. In the initial supernatant, the SCOD concentration, soluble proteins content, soluble polysaccharides content, and SCFAs concentration were 240 ± 85, 62.2 ± 12.7, 26.6 ± 5.8, and 45.7 ± 30.0 mg COD/L, respectively.

### Anaerobic Fermentation Experiment

The NaCl assistant anaerobic fermentation was performed in four batch reactors (working volume = 500 ml) and 450 ml thickened WAS was filled each. The NaCl agent (AR) was added into these batch reactors to adjust the NaCl concentrations to 0 (control), 10, 20, and 30 g/L, respectively. Then, these reactors were flushed with N_2_ and were stirred in an air-bath shaker at 120 rpm (35 ± 1°C). The sludge samples were collected on day 4 of the anaerobic fermentation for the analyses of hydrolase activity and microbial community. The sludge pH during the NaCl assistant anaerobic fermentation process was in the range of 6.7–7.0, without adjustment. The fermentation period (4 days) was selected according to our previous study, in which the sludge samples and experimental conditions were the same. In our previous study, the optimal period of NaCl assistant anaerobic fermentation was found to be 4 days (Pang et al., 2020e). The experimental results are averaged in triplicates.

### Hydrolase Activities

The protease and α-glucosidase activities were measured following the procedures in existing literatures (Liu et al., 2015). The Tris-HCl buffer (pH = 8) and Triton X-100 were employed for extracting the protease from sludge samples. The protease extraction solution was used for protease activity quantification, while the sludge samples were used for α-glucosidase activity measurement. The measurement procedure of hydrolase activity was the same for different sludge samples.

### Denaturing Gradient Gel Electrophoresis Analysis

The Fast DNA Spin Kit-EZ-10 was used to extract total DNA according to the method described in specification. After the successful extraction by gel electrophoresis, the DNA was stored in a refrigerator (−20°C) for further processing. The GeneAmp PCR System 9700 (PE9700, ABI, USA) was used to amplify 16S rRNA genes, the steps were as follows: denaturation (95°C, 30 s), denaturation (94°C, 45 s), annealing (60°C, 45 s), extension (72°C, 60 s), and extension (72°C, 600 s). Primers 338F (FACTCCTACGGGAGGCAGC) and 518R (ATTACCGCGGCTGCTGG) were used for gene amplification. Electrophoresis was carried out using buffer (1 × TAE, 80 V, 60°C, 720 min). Then, the gel was stained with SDNA-nucleic acid staining dye (Bio Basic Inc., Canada), followed by washing with sterile water and scanning using a projection scanner (PowerLook 1,000, Umax, China). Finally, the samples were sequenced on DNA sequencing system (ABI3730XL).

In order to assess the microbial community structure at different NaCl concentrations, the richness and diversity indexes were analyzed. The denaturing gradient gel electrophoresis (DGGE) band results were analyzed by Quantity One version 4.6.2 analysis software, i.e., the band numbers and strength were determined, followed by calculation according to the procedure in previous study (Polli et al., 2018). The DGGE technology is economical and dependable, which is sufficient for analyzing the difference of microbial community structure between sludge samples. In this study, the DGGE results were expected to provide some valuable conclusions.

## Results and Discussion

### Effect of NaCl Concentration on Hydrolase Activity

**Figure 1:**
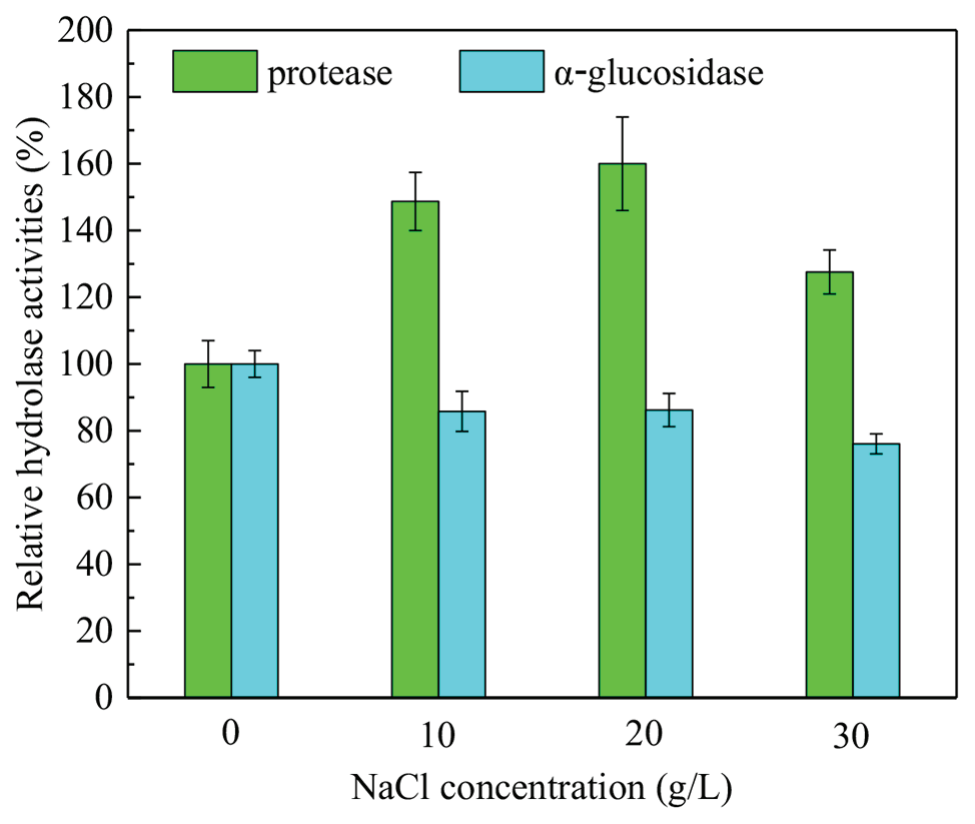
Relative activities of protease and α-glucosidase after 4-day NaCl assistant anaerobic fermentation at the NaCl concentrations of 0 (control), 10, 20, and 30 g/L.

The enzyme activity was closely related to metabolic activity of functional microorganism in sludge system, especially the hydrolase activity, which directly affects sludge hydrolysis and DOMs biodegradation in anaerobic fermentation process. During the NaCl assistant anaerobic fermentation, the additional NaCl caused remarkable osmotic pressure difference, resulting in EPS disruption and cell lysis (Pang et al., 2020d). Sludge solubilization was thereby triggered, which enhanced sludge hydrolysis and SCFAs production. Meanwhile, the high salinity environment significantly affected the survival of some microorganism, which modified microbial community structure and changed functional characteristics of sludge system. The enzyme activity was also remarkably affected. The Figure 1 presents the relative activity of hydrolase (i.e., protease and α-glucosidase) after 4-day anaerobic fermentation at different NaCl concentrations. Compared with the control test (i.e., without NaCl addition), the protease activity was significantly increased at the NaCl concentrations in range of 10–30 g/L, while the α-glucosidase activity was decreased. Overall, the presence of NaCl facilitated protease and inhibited α-glucosidase in the anaerobic fermentation system. Moreover, it was noticeable that the relative activity of protease was increased with the rising NaCl concentration from 0 to 20 g/L, afterward the protease activity gradually decreased with the further increase in NaCl concentration to 30 g/L. Apparently, 20 g/L was the optimal NaCl concentration for protease activity and hydrolysis of protein-like substances. The increasing NaCl concentration within 0–20 g/L facilitated protease secretion and improved protease activity, further increasing NaCl concentration might injure some function microbes and inhibits protease activity. In contrast, the relative α-glucosidase activity presented decrease trend with the increasing NaCl concentration. Obviously, the protease was resistant to the NaCl presence when the NaCl concentration was less than 20 g/L, whereas α-glucosidase was vulnerable to the high salinity condition. The hydrolase activity was associated with the hydrolysis efficiency of DOMs. It could be inferred that the hydrolysis of proteins was improved and the hydrolysis of carbohydrates inhibited in the NaCl assistant anaerobic fermentation.

### Functional Microbial Community Identification: DGGE Band Analysis

**Figure 2:**
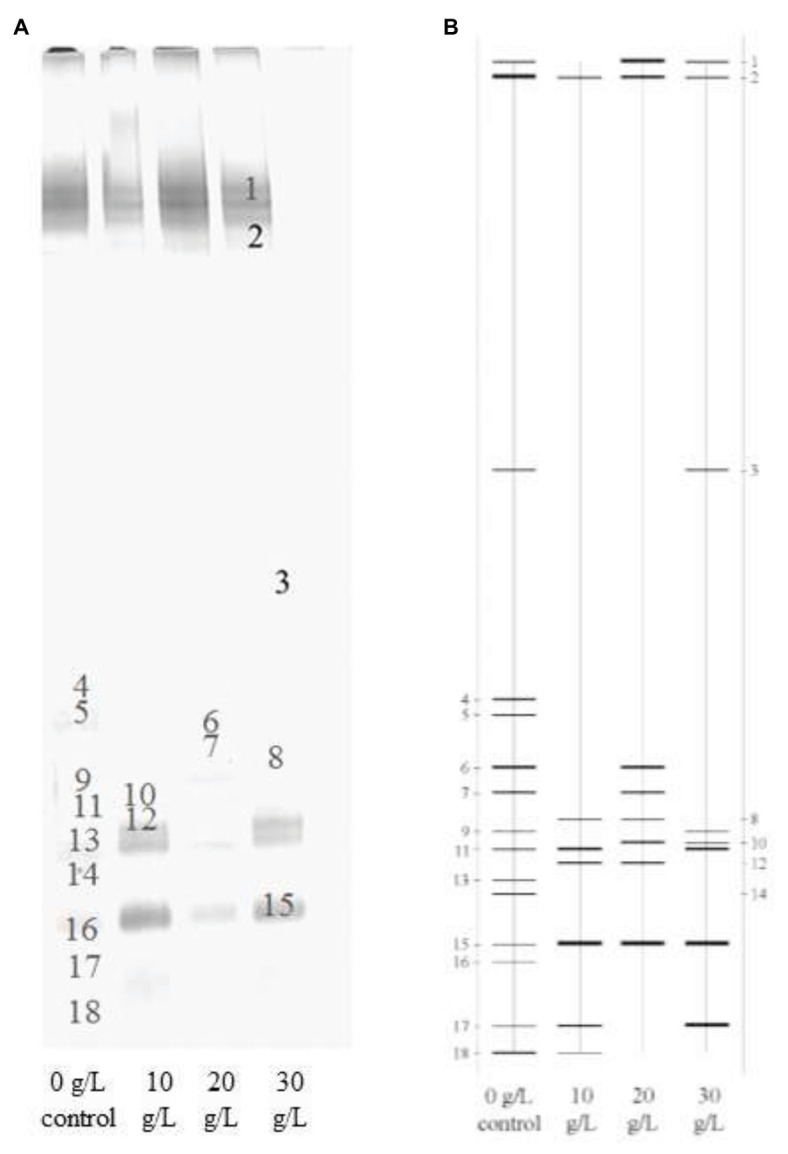
DGGE fingerprint of microbial communities at different NaCl concentrations of 0 (control), 10, 20, and 30 g/L: **(A)** DGGE profiles and **(B)** DGGE diagram.

**Figure 3:**
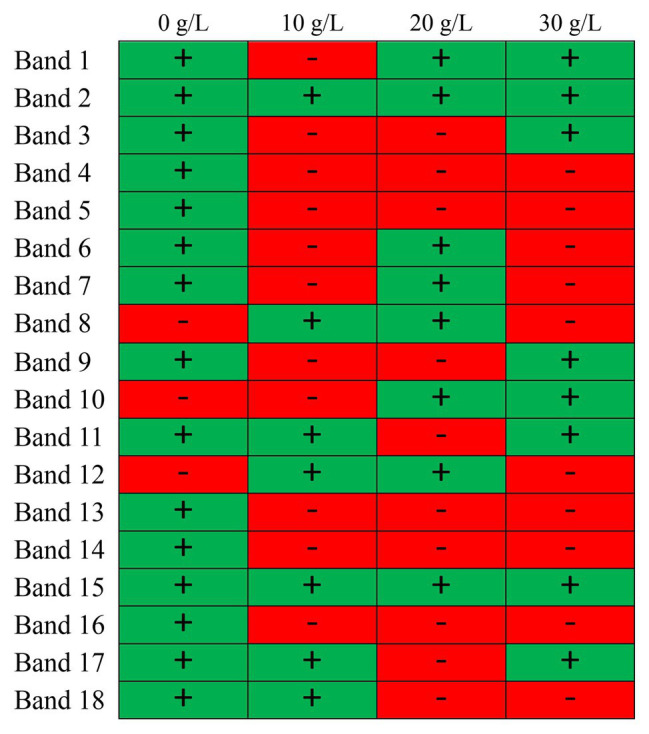
Illustration of bacteria distribution in NaCl assistant anaerobic fermentation system at different NaCl concentrations.

To explore the effects of NaCl concentration on microbial community in NaCl assistant anaerobic fermentation process, the DGGE analysis was employed. The DGGE fingerprint at different NaCl concentrations (0–30 g/L) is presented in Figure 2A, and the DGGE diagram is provided in Figure 2B for more clear demonstration. The location and lightness of the bands represent the bacteria species and abundance, respectively. Obvious differences of bacterial species between the samples at different NaCl concentrations were observed. Moreover, the color depth and the line roughness of bands could be used for assessing bacterial abundance, i.e., the deeper color in Figure 2A and the more rough line in Figure 2B were associated with the higher bacteria abundance. This implied that the relative abundances of each bacteria were diverse with different NaCl concentrations. Figure 3 displays the microbial community distribution of the sludge samples, which also proved the diverse evolution of microbial community at different NaCl concentrations. According to the band strength and bacteria distribution in Figures 2, 3, it was observed that the bacteria species were reduced with the presence of NaCl. The abundances of the bacteria related to Band 2, 4, 5, 6, 7, 9, 13, 14, 16, and 18 were reduced with the rising NaCl concentrations, while the abundances of bacteria related to Band 1, 8, 10, 11, 12, 15, and 17 were increased.

**Figure 4:**
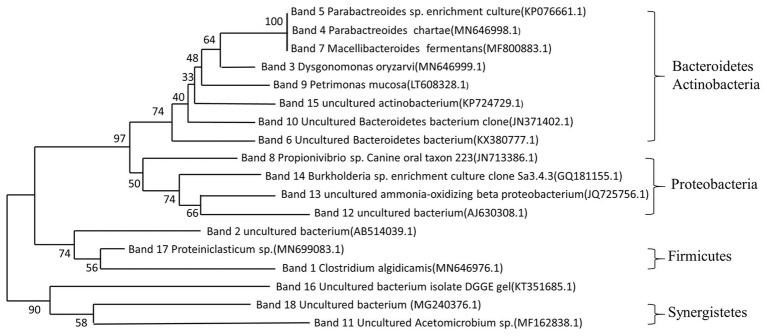
Neighbor-joining tree for phylogenetic identities of the bacteria in DGGE bands.

The bacteria identification of the bands from DGGE profile is shown in Table 1, and the main physiological functions of these functional bacteria are summarized in Table 2. Moreover, the neighbor-joining tree was analyzed for identifying the bacterial affiliation from DGGE band sequences to the database sequences as shown in Figure 4. The bacteria with closed distance in neighbor-joining tree implied that these two bacteria might be affiliated to the same phylum or class with similar functions. It can be seen in Figure 4 that the major phyla in the sludge system after 4-day anaerobic fermentation include Firmicutes, Bacteroidetes, Proteobacteria, Synergistetes, and Actinobacteria, which were reported to widely present in anaerobic digestion processes (Pang et al., 2020c). It was found that the phylum Proteobacteria was responsible for cell lysis, intracellular material release, and organic compound degradation (Kim et al., 2009), while Bacteroidetes could utilize the released intracellular material to carry out hydrolytic fermentation and possibly release proteinaceous EPS (Han et al., 2015). It was also reported that the Phylym Firmicutes includes extremely resistant microorganisms and endospores, which could also trigger the biodegradation of some organic compounds (Garcia et al., 2011).

**Table 1:** Phylogenetic affiliation of the bacteria from DGGE bands.

Bands	Nearest sequence	Accession No.	Similarity (%)	Class	Phylum
1	*Clostridium algidicarnis*	MN646976	87.23	Clostridia	Firmicutes
2	Uncultured bacterium	AB514039	100	-	-
3	*Dysgonomonas oryzarvi*	MN646999	94.81	Bacteroidia	Bacteroidetes
4	*Parabacteroides chartae*	MN646998	96.32	Bacteroidia	Bacteroidetes
5	*Parabacteroides sp. enrichment culture*	KP076661	98.61	Bacteroidia	Bacteroidetes
6	Uncultured Bacteroidetes bacterium	KX380777	90.21	-	Bacteroidetes
7	*Macellibacteroides fermentans*	MF800883	98.05	Bacteroidia	Bacteroidetes
8	*Propionivibrio sp. canine oral taxon 223*	JN713386	91.3	Betaproteobacteria	Proteobacteria
9	*Petrimonas mucosa*	LT608328	98.25	Bacteroidia	Bacteroidetes
10	*Uncultured Bacteroidetes bacterium clone*	JN371402	100	-	Bacteroidetes
11	Uncultured *Acetomicrobium sp.*	MF162838	100	Synergistia	Synergistetes
12	Uncultured bacterium	AJ630308	86.32	-	-
13	Uncultured ammonia-oxidizing beta proteobacterium	JQ725756	85.84	Betaproteobacteria	Proteobacteria
14	*Burkholderia sp. enrichment culture clone Sa3_4.3*	GQ181155	85.84	Betaproteobacteria	Proteobacteria
15	Uncultured actinobacterium	KP724729	93.68	-	Actinobacteria
16	Uncultured bacterium isolate DGGE gel	KT351685	87.5	-	-
17	*Proteiniclasticum sp.*	MN699083	93.55	Clostridia	Firmicutes
18	Uncultured bacterium	MG240376	94.57	-	-

**Table 2:** Main functions of the key bacteria.

Nearest sequence	Bands	Main functions	Specific functions	References
*Clostridium algidicarnis*	1	Fermentation bacteria for DOMs biodegradation and SCFAs production	Bio-production of SCFAs and hydrogen.	Lawson et al., 1994
*Dysgonomonas oryzarvi*	3		Biodegradation of glucose, galactose, xylose, cellobiose, lactose, sucrose, D-ribose, fructose to produce SCFAs (especially acetic acid).	Kodama et al., 2012
*Parabacteroides chartae*	4		Biodegradation of glucose, lactose, sucrose, maltose, xylose, cellobiose to produce lactic acid, propionic acid, formic acid and acetic acid.	Tan et al., 2012
*Parabacteroides sp. enrichment culture*	5			
*Macellibacteroides fermentans*	7		Biogradation of carbohydrates to produce SCFAs	Wang et al., 2019
*Propionivibrio sp. canine oral taxon 223*	8		Bio-production of SCFAs (especially acetic and propionic acids)	Brune et al., 2002
*Petrimonas mucosa*	9		Biogradation of carbohydrates to produce SCFAs	Wang et al., 2019
Uncultured *Acetomicrobium sp.*	11		Biogradation of carbohydrates to produce SCFAs	Soutschek et al., 1984
Uncultured ammonia-oxidizing beta proteobacterium	13		Ammonia oxidization, degradation of proteins and amino acids	Wittebolle et al., 2005
*Burkholderia sp. enrichment culture clone Sa3_4.3*	14		Biodegradation of lipid	Lee et al., 2014
*Proteiniclasticum sp.*	17		Biodegradation of proteins to produce SCFAs	Wang et al., 2019

Obviously, the phyla in the four sludge samples were mainly responsible for sludge hydrolysis and anaerobic fermentation. The reduced bacteria abundances might attribute to the inhibition of NaCl on bacteria growth. The inhibited bacteria were vulnerable to high salinity condition, i.e., *Parabacteroides chartae*, *Parabacteroides*, *Macellibacteroides fermentans*, *Petrimonas mucosa*, ammonia-oxidizing beta proteobacterium, and *Burkholderia*. It indicated that many bacteria in these genera were eliminated because they were more susceptible to the high NaCl concentration. Some research have reported that the major ecological function of *Parabacteroides chartae*, *Macellibacteroides fermentans*, and *Petrimonas mucosa* was the degradation of carbohydrates, which was consistent with the decreased α-glucosidase activity in Figure 1. Compared with control test (0 g/L), the genera, including *Clostridium algidicarnis*, *Propionivibrio* sp. canine oral taxon 223, uncultured *Acetomicrobium* sp., uncultured actinobacterium, and *Proteiniclasticum* sp., were enriched, which were salt-tolerant and were mostly associated with proteins biodegradation and SCFAs production. The above results revealed that the presence of NaCl during the 4-day anaerobic fermentation period caused bacteria abundance variations. The long-term exposure to high salinity condition resulted in significant reductions in some certain bacteria abundances related to carbohydrate hydrolysis, while some protein-hydrolysis bacteria and SCFAs producers were survived. The enrichment of these resistant bacteria contributed to the bio-production of SCFA and the biodegradation of proteins during the NaCl assistant anaerobic fermentation. These results indicated that the presence of NaCl changed the structural composition of microbial community, which created a good favorable environment for anaerobic fermentation. Under the condition with different NaCl concentrations, the NaCl assistant anaerobic fermentation was related to the evolution of microbial community composition. Besides, it could be inferred that the SCFAs was mainly produced from the biodegradation of proteins, while the hydrolysis of carbohydrates was inhibited. The migration and shift of microbial community would promote the efficient enrichments of protein-degrading and SCFAs-producing bacteria, resulting in the accumulation of a large amount of SCFAs during NaCl assistant anaerobic fermentation process.

Although the anaerobic microbes would be impacted when the NaCl concentration is too high, both the SCFAs production and the SCFAs-producing bacteria abundance were significantly improved in NaCl assistant anaerobic fermentation process. The anaerobic fermentation (i.e., SCFAs production) includes three steps: sludge hydrolysis, acidification, and methanogenesis. On the one hand, although the high NaCl concentration might inhibited metabolism of microbes, the sludge solubilization and hydrolysis were significantly improved, which provided more substrates for SCFAs production. On the other hand, the methanogenesis was inhibited by NaCl, which reduced SCFAs consumption and was beneficial for the anaerobic fermentation performance. The inhibition of sodium on methanogens might be greater than that on SCFAs-producing bacteria, indicating that high NaCl concentration was beneficial for SCFAs accumulation. Furthermore, the improvement of NaCl addition on SCFAs-producing bacteria was resulted from two aspects: (1) the NaCl-caused sludge solubilization provided numerous biodegradable organic matters, which facilitated the acidification step in anaerobic fermentation process and promoted the growth of SCFAs-producing bacteria and (2) the high salinity condition seriously inhibited methanogens rather than SCFAs-producing bacteria, i.e., the inhibited growth of other bacteria increased the abundances of SCFAs-producing bacteria. Similar phenomenon has been observed in previous studies (Su et al., 2016).

### Richness and Diversity of Microbial Community

According to the DGGE fingerprint and bacteria identification in Figure 2 and Table 1, phyla Bacteroidetes and Proteobacteria were mostly inhibited. It was observed that the band numbers of DGGE profiles in the presence of NaCl were less than that in the control, while the band number and location were also diverse, which implied varied microbial community richness and diversity at different NaCl concentrations. In order to explore the effects of NaCl concentration on microbial community structure, the richness, diversity, and evenness of microbial community were analyzed as shown in Table 3. The microbial community richness was used for evaluating the specie numbers in microbial community (Pang et al., 2020c), which was calculated using the bank amounts in this study. It was found that the microbial community richness was significantly reduced in the presence of NaCl, which was in the range of 7–8 at the NaCl concentrations of 10–30 g/L, while the higher richness value of 15 was observed in the control. In the anaerobic fermentation process, the sludge sample without NaCl addition has higher species abundance than those with NaCl addition. Apparently, high NaCl concentration inhibited the survival of some microbes in sludge system, i.e., the species abundance was reduced with the NaCl presence, which decreased the microbial community richness. It should be realized that the microbial community richness at the different NaCl concentrations (10–30 g/L) were similar, implying that the varying NaCl concentrations have similar inhibition effects on microbial growth and have similar species reduction performance in the anaerobic fermentation process.

**Table 3:** The richness, diversity, and evenness of microbial communities at different NaCl concentrations.

	Richness	Diversity	Evenness
0 g/L (control)	15	2.7046	0.998726
10 g/L	7	1.9372	0.995524
20 g/L	8	2.0767	0.998682
30 g/L	8	2.0754	0.998056

The Shannon and Pielou indexes could assess the diversity and evenness of the microbial community related to different NaCl concentrations (Xin et al., 2015). The relative abundance and the number of species impacted the Shannon index (Miura et al., 2007). In this study, the Shannon index and the Pielou index were calculated according to the band numbers and the relative abundance of each frequency band. It is found in Table 3 that the diversity of microbial communities at the NaCl concentrations of 10–30 g/L were much lower than that in the control, indicating the microbial community diversity was decreased owing to the inhibition of additional NaCl on the microbial species growth. Moreover, it was noticeable that the microbial community diversities at the NaCl concentrations of 20 and 30 g/L were similar, which were bother higher than that at the NaCl concentration of 10 g/L. Although the additional salinity was unfavorable for the growth of some microbes, the higher NaCl concentration might facilitate microbial activity compared with low NaCl concentration, i.e., increased NaCl concentration was beneficial for microbial community diversity when the NaCl concentration was higher than 10 g/L. The increased diversity was beneficial for the stability of microbial community structure and the comprehensive functions of sludge system. The reduction of microbial community diversity was a response of the microbial community to resist the perturbed conditions (i.e., high NaCl concentration). The release of lysed nuclear matters owing to NaCl-caused EPS disruption and cell lysis might also contribute to decrease the biodiversity of sludge bio-samples. In contrast, the evenness of microbial communities at different NaCl concentrations did not significantly differ to each other. The NaCl concentration has little effect on microbial community evenness. In consequence, the attack of additional NaCl performed a significantly negative effect on richness and diversity of microbial community, and the increased NaCl concentration (10–30 g/L) facilitated to improve microbial community diversity, while the microbial community evenness was not affected by NaCl concentration.

### The Similarity of Microbial Communities Related to Different NaCl Concentrations

**Figure 5:**
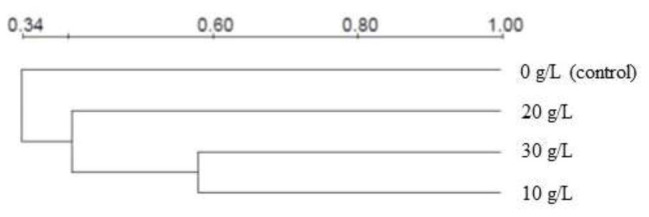
Clustering analysis for evaluating the similarity between microbial communities at different NaCl concentrations.

As shown in Table 4, the Dice coefficient was used to quantify the similarity of DGGE spectra between the microbial communities at different NaCl concentrations. It was observed that the similarity of the sludge samples with each other was low, the similarity coefficients were all below 60%, which implied that the varied NaCl concentrations triggered significant microbial community evolution. Moreover, it was noticeable that the similarity coefficients of the 10 g/L test with the 20 and 30 g/L tests were 46.5 and 58.2%, respectively, which were a bit higher than the similarity coefficients among other tests. The microbial community at the NaCl concentration of 10 g/L was a bit similar to those at 20 and 30 g/L. Similar results could be also observed by the clustering analysis (Figure 5). Apparently, the presence of NaCl significantly changed microbial community composition, whereas the microbial community evolutions between different NaCl concentrations (10–30 g/L) were less obvious. The dissimilarity of microbial communities at different NaCl concentrations might attribute to the decreased microbial community richness and diversity (Table 2). The above results suggested that (1) evolution of microbial community existed obviously with the presence of NaCl; (2) increase in NaCl concentration had a positive impact on the dissimilarity of microbial communities; and (3) the dissimilarity among the control test with the NaCl tests was the most significant, i.e., the microbial community dissimilarity with the “NaCl grow out of nothing” was more obvious than the “further increase NaCl concentration” one.

**Table 4:** The similarity coefficient (%) between sludge samples at different NaCl concentrations.

	0 g/L (control)	10 g/L	20 g/L	30 g/L
0 g/L (control)	100.0	-	-	-
10 g/L	29.2	100.0	-	-
20 g/L	39.4	46.5	100.0	-
30 g/L	32.7	58.2	34.8	100.0

### Microbial Dynamic Implications

According to the findings in this study, the hydrolytic bacteria and acidogens were enriched with the presence of NaCl, especially at the optimal NaCl concentration of 20 g/L, which facilitated the hydrolysis stage and acidification stages. In contrast, the bacteria responsible for SCFAs consumption and methane production were inhibited with decreased abundance due to the attack of high salinity condition on SCFAs-consuming bacteria, which was beneficial for reducing SCFAs consumption and facilitating SCFAs accumulation in anaerobic fermentation. Moreover, the increased protease activity and decreased α-glucosidase activity with rising NaCl concentration are observed in Figure 1. It could be inferred that the hydrolysis of protein-like substances was improved with NaCl assistance, while the hydrolysis of carbohydrates might be a bit poor. During the NaCl assistant anaerobic fermentation, the bacteria responsible for DOMs biodegradation and SCFAs production were enriched, these bacteria were resistant to high NaCl concentration. On contrary, the SCFAs consumers (e.g., methanogens, etc.) were inhibited, which were vulnerable to high salinity condition. Obviously, the external NaCl modified microbial community structure by richness, diversity, and evenness, which created a liquid environment favorable for acidogenic fermentation.

Although the optimal NaCl concentration of 20 g/L was high, the NaCl could be recovered and reused after utilization of the produced SCFAs in fermentative liquid, which reduced the treatment costs and environmental hazard. The fermentative liquid could be used as external carbon source for biological electricity production (MFC) or hydrogen production (microbial electrolysis cells). In this way, the effluent with numerous NaCl could be reused for NaCl assistant anaerobic fermentation process.

## Conclusion

This study demonstrated that NaCl could modify hydrolase activity and microbial community structure in 4-day anaerobic fermentation. With the rising NaCl concentrations (0–30 g/L), the protease activity was increased to 127.6–160%, while the α-glucosidase activity was reduced to 76.1–86.2%. Some carbohydrate-hydrolysis bacteria (e.g., *Parabacteroides chartae*, *Macellibacteroides fermentans*, and *Petrimonas mucosa*) were inhibited by high salinity condition, while the protein-degrading and SCFAs-producing bacteria were resistive and enriched. Compared with control test, the microbial community richness and diversity were reduced in NaCl test (10–30 g/L), while the evenness was almost changeless. The similarity between communities was low. Moreover, the pathway of DOMs biodegradation and SCFAs production in NaCl assistant anaerobic fermentation was illustrated.

## Data Availability Statement

The raw data supporting the conclusions of this article will be made available by the authors, without undue reservation.

## Author Contributions

HP: writing-original draft and data curation. XX: investigation. ZY: methodology. BC: writing-original draft. CL: data curation. XW: software. DG: writing-original draft, conceptualization, and formal analysis. JH: supervision. JN: conceptualization. All authors contributed to the article and approved the submitted version.

### Conflict of Interest

CL was employed by the company Frog Biotechnology Co., LTD, Harbin, 350116, China.

The remaining authors declare that the research was conducted in the absence of any commercial or financial relationships that could be construed as a potential conflict of interest.
